# Examining the Relationship between Psychological Functioning, Childhood Trauma, and Types of Perceived Coercion Among Drug Court Enrollees: Results from A Pilot Study

**DOI:** 10.1080/23311908.2017.1320859

**Published:** 2017-05-04

**Authors:** Andrea N. Cimino, Natasha Mendoza, Thomas H. Nochajski, Mark G. Farrell

**Affiliations:** Faculty Research Associate, Johns Hopkins University, School of Nursing, The State University of New York; Assistant Professor, Arizona State University, School of Social Work, The State University of New York; Research Professor, Buffalo Center for Social Research, University at Buffalo, The State University of New York; Judge, Amherst Town Drug Court, Amherst, NY

**Keywords:** substance abuse, drug treatment, court ordered, mandated populations, trauma-informed practice

## Abstract

**Background:**

Drug court interventions produce positive results—especially among mandated populations. Many criminal justice-involved persons, including drug court enrollees, have cooccurring substance abuse and childhood trauma disorders associated with psychological dysfunction. Given the coercive nature of mandated drug court treatment, it is important to understand whether childhood trauma and psychological functioning influence perceived coercion to enter treatment.

**Objectives:**

The purpose of this study was to describe the degree to which adverse childhood trauma and psychological functioning were associated with six domains of perceived coercion— self, family, legal, financial, health, and work—among a population of drug court enrollees.

**Methods:**

Data from 54 enrollees in a drug court pilot study were used to examine the relationship between childhood trauma, psychological functioning, and perceived coercion.

**Results:**

The pilot study data showed that psychological dysfunction and traumatic experiences in childhood were associated with higher perceived coercion to treatment, explaining 29% of the variance in coercion, controlling for gender and pre-arrest alcohol and drug use. Results indicated that the associations between psychological dysfunction and trauma were driven by non-legal types of coercion. In particular, childhood trauma was correlated with family (*r* = .32), financial (*r* = .32), and health (*r* = .47) types of coercion.

**Conclusions:**

Based on these preliminary findings, drug court practitioners are urged to assess perceived coercion, in addition to the behavioral health and childhood trauma of their clients, and to utilize non-legal types of coercion such as family, health, and financial impact to enhance treatment engagement.

## Introduction

Drug court is a criminal justice intervention targeting individuals who abuse alcohol and drugs. In the United States (U.S.), arrests may or may not be the result of an alcohol or drugrelated offense (depending on the jurisdiction); however, individuals are determined by the court to have an alcohol or drug-related problem. Once participants have agreed to take part in a drug court intervention, they are typically mandated to receive a behavioral health assessment followed by court-supervised treatment for substance use disorder (National Association of Drug Court Professionals [NADCP], 2017). According to NADCP, individual participants are provided with treatment and other services to maintain sobriety, held accountable by drug court staff, regularly tested for substance use, required to appear in court regularly, rewarded for doing well and sanctioned for not following through with their obligations. Minimum terms are 1 year.

Drug court treatment may be legally mandated, whereby participants incur consequences if they refuse to comply, or non-mandated where attendance is voluntary (Polcin & Greenfield, 2003). Court-mandated treatment is popular because of its effectiveness with respect to recidivism, improved behavioral health outcomes (Mitchell et al., 2011; Shaffer, 2011), and reduced costs (Rosenberg & Mark, 2011; Roman, 2013). Meta-analyses show that enrollees mandated to drug court treatment in the United States (U.S.) have significantly lower recidivism, less substance abuse over time, and improved social functioning than non-drug court enrollees (Mitchell et al., 2011; Shaffer, 2011).

While many believe that court-mandated treatment offers the external motivation to comply that some criminals intrinsically lack, others find it coercive and unethical (Miller & Flaherty, 2000; Parhar, Wormith, Derkzen, & Beaugard, 2008). Legal coercion can be a powerful motivator to seek treatment, but non-legal types of coercion, such as pressure from family, friends, or employers also contribute to an individual’s decision to enter treatment (Wild, Roberts, & Cooper, 2002). It is not well understood how non-legal types of coercion impact treatment outcomes, particularly when there is a history of childhood trauma and psychological dysfunction, two phenomena associated with criminal justice-involved populations (Grella, Lovinger, & Warda, 2013; Zlotnick et al., 2003). The purpose of this study was to describe the degree to which childhood trauma and psychological functioning were associated with six domains of perceived coercion—self, family, legal, financial, health, and work—among a population of drug court enrollees.

Regardless of the legal context, confrontation and coercion are key to understanding one’s motivation to seek drug treatment. *Confrontation* refers to evidence that “bad things” such as loss of relationships, eviction, or jail will happen if one continues to use substances (Polcin, 2009). *Coercion* is similar except that the person making the threat has the power to implement it. For example, a spouse may confront a substance-abusing individual with the threat that he/she will go to jail, but only a probation officer or judge has the power to enact that threat. Hence, legal coercion is thought to be a more powerful motivator for treatment. Equating a legal mandate as synonymous with coercion may be too simplistic; in truth, individuals entering treatment are subject to a host of inter- and intrapersonal pressures from family, friends, employers, and other non-legal types (Klag, Creed, & O’Callaghan, 2006). For example, Mackain and Lecci (2010) found that internal coercion (e.g., “You lost respect for yourself”) was more impactful from a client’s perspective, whereas clinicians perceived external coercion (e.g., “You could lose custody of your children”) as more impactful. Differences in what a client versus a clinician or the legal system views as an important reason to seek treatment could influence the treatment approach and the therapeutic alliance (Parhar, Wormith, Derkzen, & Beaugard, 2008). Ultimately, the source of one’s motivation, and whether a person feels it is coercive, may impact treatment outcomes (Parhar, Wormith, Derkzen, & Beaugard, 2008).

### Purpose of the Study

Given the high rates of childhood trauma and co-occurring substance abuse among persons in the criminal justice system (Grella, Lovinger, & Warda, 2013; Danielson et al., 2009; Huddleston & Marlowe, 2011), it is important to understand whether childhood trauma influences coercion to treatment. Little research has examined the degree to which childhood trauma is related to perceived coercion—an important factor in motivation—in mandated populations. Our study examined whether childhood trauma and psychological functioning were associated with perceived coercion in a population of drug court enrollees. We hypothesized: (1) there would be a positive relationship between types of perceived coercion, childhood trauma, and psychological functioning, and (2) that childhood trauma and psychological functioning would explain the variance in perceived coercion, controlling for gender and pre-arrest alcohol and other drug use (AOD) among drug court enrollees.

## Methods

### Procedure and Sample

Data for the current analyses were taken from a drug court enrollee pilot study (the “parent study”). The drug court was located in a suburb of Buffalo, NY. Recruitment took place weekly over one year during drug court proceedings. Research staff privately screened potential participants to determine eligibility. To be eligible, participants had to be within one month of their referral to the current drug court, age 18 or older, understand English, and report problematic substance use (measured via Drug Abuse Screening Test [Skinner, 1982] and the Alcohol Use Disorders Identification Test [Saunders et al., 1993]). Participation in the study was not linked to participants’ the involvement in drug court and had no bearing on their open case. Moreover drug court staff, including the judge had no information about participants. IRB approval was granted by [University, blind for review]. Participants were compensated with a $40 gift card. Of the 80 individuals screened, 55 were eligible. Due to missing data, one participant was excluded, bringing the final sample size to 54. Table 1 presents participant demographics. The current study includes only data that were collected at baseline. Participants were followed for a period of three months; however, due to substantial attrition, follow-up data are untenable. A full description of the parent study and methods is provided elsewhere (Mendoza, Linley, Nochajski & Farrell, 2013).

### Measures

The 30-item *Perceived Coercion Questionnaire* (PCQ; Klag, Creed, & O’Callaghan, 2006) measures internal and external perceived coercion to enter drug and alcohol treatment across six domains: (1) *self*: I’m sick and tired of losing everything (α=.84); (2) *family*: my family made me feel guilty by telling me how much I have hurt them (α=.94); (3) *legal*: I didn’t want to do time in jail (α=.60 [see Limitations section]); (4) *financial*: I have no money to support myself (α=.90); (5) *health*: I have had enough of being sick all the time (α=.80); (6) *work*: my employer threatened to sack me (α=.90). Each item began with the statement, “I felt pressured to enter this drug/alcohol treatment program because…” Participants responded with how strongly they agreed (5) or disagreed (1) with each statement, with higher scores indicating greater coercion. We used the total score (range 30–150, α=.93) and subscale scores, which were the average of the respective items.

The *Adverse Childhood Experiences* (ACE; Felitti et al., 1998) is a 10-item instrument assessing abusive (physical, sexual, psychological) or dysfunctional childhood experiences (substance abuse, mental illness, violence). Participants received a 1 (“yes”) indicating that they experienced that adverse event (range 0–10; α=.80).

The *Global Severity Index* (GSI) is part of the *Brief Symptom Inventory* (BSI; Derogatis, 1975), a 53-item instrument assessing nine psychological dimensions: somatization, obsessioncompulsion, interpersonal sensitivity, depression, anxiety, hostility, anxiety, paranoia, and psychoticism. Participants responded with how bothered they were on a scale of “not at all” (0) to “extremely” (4). GSI score is the average of all subscales (α=.94).

Pre-arrest AOD was assessed using the following question: “Looking back at the three months prior to your last arrest, how many days per week did you drink alcohol or use drugs during this period?”

### Analysis

First, we examined data for outliers, normality, and multicollinearity, which were all adequate. Then, we examined bivariate correlations between each of the PCQ subscales, ACE, and GSI scores with a significance level set at .05. To address hypothesis 2, we conducted a hierarchical multiple regression with the PCQ total score as the dependent variable. In the first model, we entered our control variables (i.e., gender, pre-arrest AOD); in the second model, we entered ACE total score; in the final model, we entered the GSI score. All analyses were completed using SPSS 23.

#### Power.

**Figure 1.**
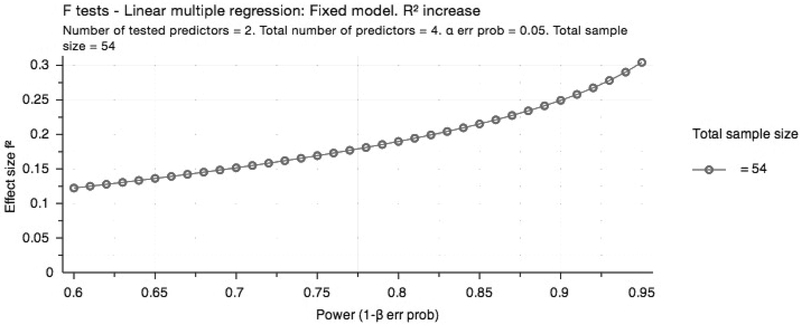
Sensitivity Power Analysis

Given that the sample size was known, (*N*=54), we conducted a sensitivity power analysis to determine the minimum effect size our regression analysis could detect. A sample size of 54 was adequate to detect effects ≥.19, with power (1-β) at .80, α=.05, and 4 predictors (see Figure 1).

## Results

Hypothesis 1: Significant, positive correlations were found between ACE and coercion from family (*r*=.32), financial (*r*=.32), and health (*r*=.47). GSI was positively correlated with coercion from oneself (*r*=.37), financial (*r*=.43), health (*r*=.34), and work (*r*=.32). Legal coercion did not have a significant relationship to ACE (*r*=.19) or to GSI (*r*=.03); however, legal coercion was the least reliable. See Table 2 for details.

Hypothesis 2: The overall regression model explained 29% of the variance in perceived coercion (*F*(4, 48)=4.96, *p*<.01). In this sample, ACE and GSI scores were associated with higher perceived coercion (β =.32, *p*<.01 and β =.26, *p*<.05, respectively), controlling for gender and pre-arrest AOD. ACE scores accounted for a significant proportion of variance after controlling for gender and pre-arrest AOD, Δ*R*^2^ = .15, *F*(1, 49)=10.67, *p*<.01. BSI scores accounted for a significant proportion of variance, after controlling for ACE, gender, and prearrest AOD, Δ*R*^2^ = .06, *F*(1, 48)=3.82, *p*<.05. See Table 3 for details.

## Discussion

While preliminary, our findings point to critical implications for research and practice. The link between trauma and perceived coercion in this population must be further explored so that clinicians better tailor motivational techniques to the needs of their mandated clients. Our analyses revealed that childhood trauma and psychological functioning were significantly related to non-legal types of perceived coercion to enter drug court treatment. Health coercion had the strongest relationship to childhood trauma (*r*=.47), which is interesting to note because adverse childhood events are predictive of later life health issues (Felitti et al., 1998). Furthermore, health and financial coercion were correlated to both childhood trauma and psychological functioning, which could indicate that these types of coercion are related—as one’s health is affected by childhood trauma, one’s finances could also be impacted, which seems to be supported by the correlation between work coercion and psychological functioning.

Relatedly, psychological functioning has been empirically linked to substance use outcomes among drug court enrollees. For example, Clark and Young (2009) conducted a national study examining the influence of coercive status (mandated vs. voluntary) and treatment condition (integrated mental health, substance abuse, and trauma treatment vs. services as usual) on psychiatric distress, trauma-related symptoms, and substance use. Results demonstrated that women did better with integrated treatment and mandated treatment regardless of their condition related to distress, trauma, or substance use.

## Limitations

We note some limitations of the study. First, we focused on baseline, cross-sectional data; thus, we cannot say that perceived coercion alone is driving one’s decision to enter treatment, nor were we able to attribute perceived coercion to treatment outcomes among this group of enrollees. Research shows that other factors, such as treatment duration and monitoring, collaboration between treatment providers, and the use of evidence-based interventions (Leffingwell, Hendrix, Mignogna, & Mignogna, 2008) are important considerations in the decision to enter and be successful in drug court treatment. Another limitation is related to potential bias associated with data collection. Participants took the survey during drug court proceedings; despite confidentiality assurances in the informed consent process, they may not have believed the data were outside of the court’s purview. Given this potential bias, it is notable that reliability for the *legal* subscale is less than other scales; while we cannot be certain, participants may have perceived coercion but did not attribute much of it to the legal system. Another limitation of the study was the small sample size, however, our sensitivity power analysis demonstrated that we were able to detect small effects.

## Conclusions

Based on these preliminary findings, drug court practitioners are urged to assess the types of perceived coercion to enter treatment, in addition to the behavioral health and childhood trauma of clients entering treatment. While the pilot sample size is a limitation, our findings suggest a need for trauma-informed practices combined with motivational enhancement techniques. Ultimately, findings support the importance of assessing trauma in mandated populations. The assessment of coercion can help practitioners more fully understand why individuals may terminate treatment early, and help explain treatment outcomes (Klag, Creed, & O’Callaghan, 2006).

## Figures and Tables

**Table 1:** Participant Demographics (N = 54)

Variable	*M*(*SD*)	*n* (%)
Age, in years	33.98(10.3)	
Gender		
Men		34 (63)
Women		20 (37)
Race		
Caucasian/White		33 (61.1)
Black/African American		19 (35.3)
Hispanic		3 (5.5)
Asian		1 (1.8)
Other Race		2 (3.7)
Marital Status		
Married		6 (10.9)
Cohabitating		3 (5.5)
Single		30 (54.5)
Separated		6 (10.9)
Divorced		9 (16.4)
Widowed		1 (1.8)
Education		
Did not graduate		9 (16.9)
Graduated/GED		22 (41.5)
Some college		14 (26.4)
College graduate		5 (9.4)
Missing		2(3.7)
Employment		
Full time		11(20.8)
Part time		7 (13.2)
Student		1 (1.8)
Unemployed		28 (52.9)
Disabled		6 (11.3)
Missing		2 (3.7)
Perceived Coercion Questionnaire (PCQ) Total	56.93(22.63)	
Self	9.68(5.63)	
Family	9.91(6.15)	
Legal	12.35(3.49)	
Financial	9.07(5.91)	
Health	9.09(5.22)	
Work	6.81(5.40)	
Adverse Childhood Events (ACE)	2.18(2.19)	
Global Severity Index (GSI)	.84(.71)	
Frequency of AOD in days per week	4.66(2.44)	

**Table 2.** Bivariate Correlation Results Between Perceived Coercion, ACES and Psychological Functioning

	PCQ-Self	PCQ-Family	PCQ-Legal	PCQ-Financial	PCQ-Health	PCQ-Work
*ACE Score*	.14	.32^*^	.19	.32^*^	.48^**^	.27
*GSI Score*	.37^**^	.12	.03	.43^**^	.36^**^	.32^*^

* *p* < .05,

** *p* < .01

**Table 3.** Regression Results Showing Variance in Perceived Coercion Uniquely Explained by Trauma and Psychological Functioning

Variables included in model	*R*^*2*^	Adj. *R*^*2*^	Δ*R*^*2*^	*B (SE B)*	β
*Model 1: Gender, Pre-arrest AOD*	.07	.03	--		
*Model 2: Gender, Pre-arrest AOD, ACE*	.24	.19	.17^**^		
*Model 2: Gender, Pre-arrest AOD, ACE, GSI*	.29	.23	.06^*^		
Intercept				30.63 (7.73)	--
Gender				7.99 (5.75)	.17
*Pre-arrest* AOD				1.53 (1.16)	.16
ACE				3.23 (1.34)^**^	.32
GSI				8.21 (4.20)^*^	.26

* *p* < .05,

** *p* < .01

*Note*: Pre-arrest AOD = pre-arrest alcohol and other drug use, ACE = Adverse childhood Experiences, GSI = Generalized Severity Index.

## Public Interest Statement

Drug court is a popular intervention for criminal justice-involved individuals who abuse alcohol and drugs. Because drug court is often mandated as part of one’s sentencing, it has been critique as coercive. Given the high rates of childhood trauma among persons in the criminal justice system as well as the co-occurrence of substance abuse, it is important to examine whether drug court enrollees feel coerced to enter drug court, as perceived coercion may be linked to treatment outcomes. This article explores the role childhood trauma and psychological functioning are related to six types of perceived coercion (self, family, legal, financial, health, work) to enter drug court. Understanding one’s perceived coercion is key to better serving this population and tailoring motivational techniques to the needs of mandated clients.
